# The Impact of Serum Oestradiol and Progesterone Levels on Intraocular Pressure and Systemic Health: Hormonal and Physiological Interactions in Female Dogs

**DOI:** 10.1002/vms3.70904

**Published:** 2026-03-24

**Authors:** Candemir Özcan, Tarik Safak, Ali Risvanli

**Affiliations:** ^1^ Department of Surgery Faculty of Veterinary Medicine Kastamonu University Kastamonu Türkiye; ^2^ Department of Obstetrics and Gynecology Faculty of Veterinary Medicine Kastamonu University Kastamonu Türkiye; ^3^ Faculty of Veterinary Medicine Kyrgyz‐Turkish Manas University Bishkek Kyrgyzstan; ^4^ Department of Obstetrics and Gynaecology Faculty of Veterinary Medicine Fırat University Elazig Türkiye

**Keywords:** dogs, hormonal influence, intraocular pressure, oestradiol, progesterone

## Abstract

**Objective:**

We hypothesized that variations in oestradiol and progesterone concentrations are significantly correlated with intraocular pressure (IOP) in female dogs and that this association is influenced by systemic physiological parameters such as age, body weight and blood pressure (BP).

**Animal studied:**

This study was performed on 37 female dogs, aged between 1 and 7 years (median: 2.5 years, range: 1–7 years), with a mean weight of 23.15 ± 9.72 kg.

**Procedures:**

The relationships between right intraocular pressure (RIOP) and left (LIOP) with systolic and diastolic BP, serum oestradiol (E2) and progesterone (P4) levels were assessed. Additional factors, such as age, pulse and body temperature, were also analysed for their correlations with IOP and hormonal levels.

**Results:**

Weak and insignificant positive correlations were noted between E2 and RIOP (*r* = 0.112, *p* = 0.255) and P4 and RIOP (*r* = 0.098, *p* = 0.283). No significant relationships were identified between E2, P4 and LIOP (E2: *r* = 0.242, *p* = 0.112; P4: *r* = 0.020, *p* = 0.460). A moderate positive association was identified between systolic BP and LIOP (*r* = 0.437, *p* = 0.003), whereas a slight positive correlation was noted between systolic BP and RIOP (*r* = 0.282, *p* = 0.045). Substantial negative associations were identified between P4 and systolic BP (*r* = −0.445, *p* = 0.010) as well as diastolic BP (*r* = −0.489, *p* = 0.005). A strong correlation was found between body weight and RIOP (*r* = 0.591, *p* < 0.001) as well as LIOP (*r* = 0.408, *p* = 0.006).

**Conclusion:**

This preliminary study highlights that reproductive hormones like E2 and P4, along with factors such as age and body weight, significantly impact physiological parameters, including heart and ocular health in female dogs. Thus, variations in reproductive hormones and factors like age and body weight should be considered when evaluating ocular health in female dogs.

## Graphical abstract

• Serum oestradiol and progesterone levels were evaluated in relation to intraocular pressure in female dogs.

• Systemic physiological parameters, particularly body weight and systolic blood pressure, showed positive associations with intraocular pressure.

• Progesterone levels were negatively correlated with blood pressure.

• These findings indicate that intraocular pressure in female dogs is more strongly influenced by systemic physiological factors than by reproductive hormone variations.

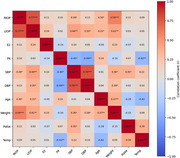

## 1 Introduction

Intraocular pressure (IOP) results from the equilibrium between the production and drainage of aqueous humour in the eye. The central nervous system efficiently maintains IOP by sustaining an ideal equilibrium between the production and drainage of aqueous humour (Okur et al. 2023). Increased IOP is a key risk factor for glaucoma prevention, with fluctuations in IOP being closely and significantly linked to variations in blood pressure (BP) (Nirmala et al. 2014).

During the menstrual cycle, complex hormonal alterations occur, influencing vascular physiology and inducing haemodynamic variations across distinct phases of the cycle (Mills, Nelesen, et al. 1996; Mills, Ziegler, et al. 1996). The sexual cycle in dogs is usually divided into three main phases: proestrus, oestrus and metestrus. During these different periods, there are significant changes in oestradiol (E2) and progesterone (P4) levels (Rowland et al. 2023). It is stated that E2 may decrease BP by increasing systemic vasodilation (Harvey et al. 2015). However, P4 has been reported to have both vasodilatory (Shi et al. 2023) and vasoconstrictive (Childs et al. 2010) effects.

Variations in systemic BP are closely linked to changes in IOP, highlighting the importance of systemic vascular health in maintaining ocular perfusion and overall ocular health (Hartmann et al. 2023). Additionally, a positive correlation between SBP and IOP has been observed, with studies reporting normal IOP values in healthy dogs ranging from 10 to 21 mmHg, influenced by health status and measurement techniques (Wrześniewska et al. 2018; Jürgens et al. 2012).

Hormones, such as E2 and P4, are known to affect the production and drainage of aqueous, which is crucial for maintaining normal IOP. The ciliary processes, responsible for aqueous humour production, and the drainage channels are sensitive to hormonal fluctuations, which can lead to variations in IOP (Saylik and Saylık 2014; Sebbag 2023). Furthermore, there is a relationship between systemic BP and ocular perfusion pressure, noting that changes in SBP can significantly affect IOP in patients with glaucoma (Jürgens et al. 2012). This relationship is critical, as maintaining adequate ocular perfusion is essential for the health of the optic nerve and overall eye function. Changes in SBP are associated with changes in IOP, reinforcing the idea that systemic vascular health is closely linked to ocular health (Hartmann et al. 2023).

The ageing process can lead to changes in IOP by affecting fluid dynamics in the eye (Olusanya et al. 2022). As the ageing process causes many physiological changes, these changes also affect IOP. In humans, there are studies stating that IOP decreases with age (Yamamoto et al. 2008), as well as statements that IOP increases with age (Jeelani et al. 2014).

We hypothesized that serum E2 and P4 concentrations are significantly associated with IOP in female dogs and that this relationship is modulated by physiological parameters such as age, body weight and BP.

## 2 Materials and Methods

### 2.1 Animals

The material for the study consisted of dogs brought to Kastamonu University, Faculty of Veterinary Medicine, Department of Obstetrics and Gynaecology for examination. The study comprised 37 female dogs. The dogs were aged 2.5 years (range: 1–7 years), with a mean weight of 23.15 ± 9.72 kg. Dogs were screened through physical examination, owner‐provided medical history and routine haematological and biochemical tests to exclude underlying illnesses. Ocular assessments included fluorescein staining and indirect ophthalmoscopy. To minimize bias, general clinical, reproductive and ophthalmic examinations were each performed by different specialists. The dogs with the proper medical history, physical examination and disposition for the study were relocated to the examination room to measure body temperature (°C). Thus, it was aimed to make accurate digital and thermal measurements by allowing the animals sufficient time to acclimate to the environment, thereby minimising stress variables that may affect the results.

Inclusion criteria required intact, non‐pregnant female dogs deemed clinically healthy based on physical examination and routine haematological and biochemical analyses. Dogs with chronic illnesses, gastrointestinal disorders, acute infections, severe environmental stress or uterine pathologies such as pyometra, mucometra or hydrometra, as detected by ultrasonographic examination, were excluded from the study. Additionally, a thorough clinical examination was performed by a veterinary surgeon to rule out conditions that could affect IOP, including epiphora, excessive lacrimation, ocular discharge, blepharospasm, entropion, keratitis, lens luxation and corneal erosion.

### 2.2 Physiological Parameter Monitoring

The SBP and DBP, pulse and respiration rate were all measured using a patient monitor (Comen C80‐V, Shenzhen, China). Electrocardiogram leads were attached to the front and left hind legs, and data were acquired. The animals included in this study did not require sedation for these evaluations. For each parameter (BP, pulse and respiration), three consecutive readings were recorded, and the average was used in the final analysis.

### 2.3 IOP Measurement

The IOP was measured using a rebound tonometer (TonoVet Plus, Jorgensen Laboratories, Loveland, CO, USA). Tonometry has the ability to calibrate itself before each use. The IOP measurements were conducted by one examiner, positioned at a 90° angle to the central cornea. IOP was initially measured from the right eye in each dog. The tonometer recorded six successive readings per measurement. IOP measurements were performed without topical anaesthesia. Dogs were gently restrained in a seated position. All measurements were conducted between 9:00 and 11:00 AM to minimize diurnal variability.

### 2.4 Measurement of Rectal Temperature

A digital thermometer that had been calibrated previously (Albert KERBL, GmbH, Germany) was utilized for each measurement. The probe was inserted approximately 1–3 cm into the rectum, depending on the size of the dog, consistent with standard clinical practice. The device measures temperatures from 32.0°C to 42.0°C, offering a precision of ±0.1°C. This rectal thermometer offers a quick response time of approximately 9–11 s and emits an audible alert when the maximum temperature is attained. After the signal from the thermometer was detected, it was taken out of the rectum, and the data were then recorded on the display screen. The probe of the digital thermometer was coated with glycerine prior to its insertion into the rectum. Temperature was recorded in a quiet room maintained at 22–24°C. All dogs were calm, and measurements were taken between 9:00 and 11:00 AM to ensure consistency (Ozcan et al. 2025).

### 2.5 Ocular Examination

To assess the presence of erosion on the ocular surface of the dogs, an exam known as fluorescein sodium staining (Fluorescite Ophthalmic Disclosing Agent Fluorescein Sodium 10%) was conducted.

### 2.6 Ultrasonographic Evaluation of the Genital Organs

The ultrasound evaluation was performed in accordance with the protocols described by Safak et al. (2021). A 5 MHz convex transducer in B‐mode (Versana Active, USA) was employed for transabdominal ultrasonography to diagnose pregnancy in bitches. Although microconvex probes offer greater anatomical accessibility, the convex probe provided adequate visualization of the uterus and ovaries in the medium‐sized dogs included in this study. The examination was conducted with the animals positioned in a ventro‐dorsal orientation, enabling visualization of the ovaries and uterus.

### 2.7 Hormone Analysis

Approximately 3 mL of blood was collected from each dog and transferred into gel serum tubes (BD, Plymouth, UK) containing a clot activator. The blood samples were centrifuged at 2000 *g* for 10 min to separate the serum for biochemical analyses. The obtained serum samples were stored at −20°C until analysis of E2 and P4 levels. After thawing, the concentrations of E2 and P4 were measured using enzyme‐linked immunosorbent assay (ELISA) kits (Bioassay Technology Laboratory, Shanghai, China).

The ELISA procedure was performed according to the manufacturer's instructions and established protocols in the literature (Safak and Risvanli 2022). Following the completion of all assay steps, E2 and P4 concentrations in the samples were determined by measuring absorbance at 450 nm using an ELISA reader (Bio Tek Instruments, USA).

### 2.8 Statistical Analysis

**FIGURE 1:**
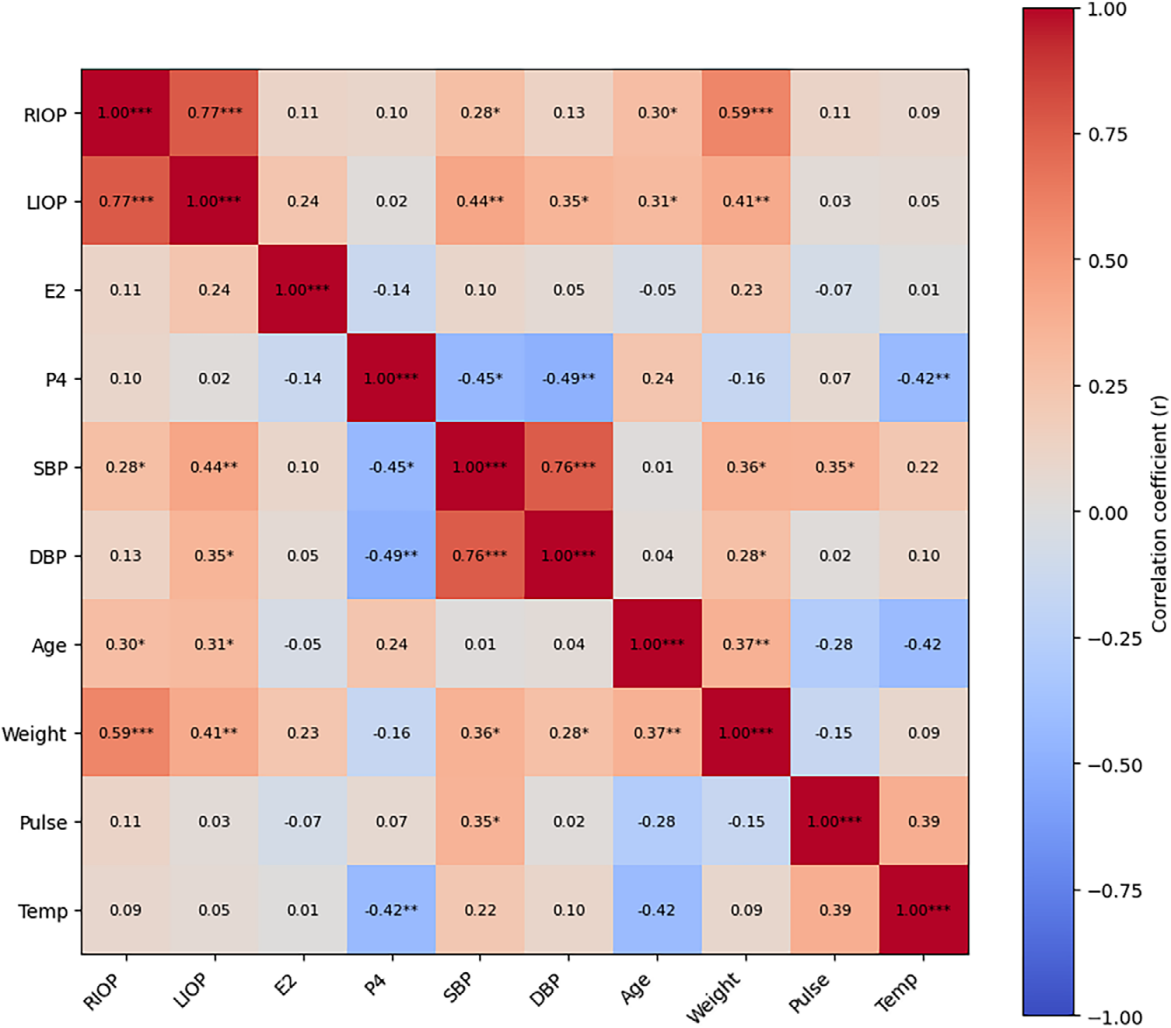
Heat map of the correlation matrix showing the relationships between right and left intraocular pressure, hormonal values and systemic physiological parameters. Warm colours (red) represent strong positive correlations, whereas cool colours (blue) indicate low or negative correlations. DBP, diastolic blood pressure; E2, oestradiol; LIOP, left eye intraocular pressure; P4, progesterone; RIOP, right eye intraocular pressure; SBP, systolic blood pressure; Temp, body temperature.

The normality of the data distribution was assessed using the Shapiro–Wilk or Kolmogorov–Smirnov tests. Variables with 𝑝 > 0.05 were considered to follow a normal distribution, and Pearson correlation analysis was applied to evaluate the relationships between these variables. For variables with 𝑝 ≤ 0.05, indicating a non‐normal distribution, Spearman rank correlation analysis was used. Statistical significance was set at 𝑝 < 0.05. Analyses were performed using SPSS 23 software. Descriptive statistics of the variables are given in Table 1. A heat map plot of the correlation matrix shows the relationships between variables, with warm colours (red) indicating high positive correlations and cool colours (blue) representing low or negative correlations (Figure 1).

**TABLE 1:** Descriptive statistics of physiological, hormonal and demographic parameters in female dogs.

	Age	Weight (kg)	Pulse (bpm)	Temp. (°C)	E2 (pg/mL)	P4 (ng/mL)	RIOP (mmHg)	LIOP (mmHg)	SBP (mmHg)	DBP (mmHg)
*n*	37	37	37	37	37	37	37	37	37	37
Mean	2.92	23.15	109.27	39.1027	129.37	9.04	25.11	25.08	149.92	91.97
Std. deviation	1.46	9.71	30.02	0.62	87.91	1.95	6.92	6.35	27.92	19.67
Minimum	1	5	36	37.8	48.89	5.4	14	13	93	54
Maximum	7	45	182	40.2	418.76	14.45	42	37	240	135
Median	2.5	20	112	39.2	90.43	9.01	23	24	150	90
Shapiro–Wilk W	0.831	0.952	0.989	0.95	0.719	0.923	0.937	0.971	0.957	0.981
Shapiro–Wilk p	<0.001	0.114	0.975	0.11	<0.001	0.014	0.038	0.441	0.159	0.750

*Note*: n: number of cases; Temp: body temperature; E2: oestradiol; P4: progesterone; RIOP: right eye intraocular pressure; LIOP: left eye intraocular pressure.

Abbreviations: DBP: diastolic blood pressure; SBP: systolic blood pressure.

## 3 Results

**FIGURE 2:**
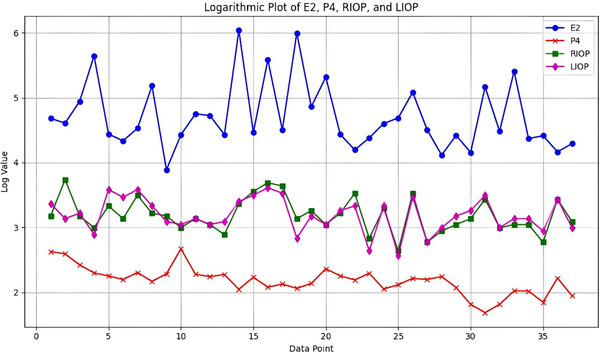
This figure shows a graph comparing the logarithmically scaled values of E2, P4, RIOP and LIOP measurements for individual dogs across different data points. Each point represents an individual animal's result. LIOP, left intraocular pressure.

A weak positive correlation was found between right intraocular pressure (RIOP) and serum E2 (*r* = 0.112, *p* = 0.255) as well as P4 (*r* = 0.098, *p* = 0.283), both of which were statistically insignificant. Significant correlation was not observed between left intraocular pressure (LIOP) and E2, nor between LIOP and P4. The relationship between E2 and LIOP was found to be low and statistically insignificant (*r* = 0.242, *p* = 0.112). Additionally, a weak correlation was noted with P4 (*r* = 0.020, *p* = 0.460). Figure 2 compares the logarithmically scaled values of E2, P4, RIOP and LIOP to enhance data stability and clarity.

**FIGURE 3:**
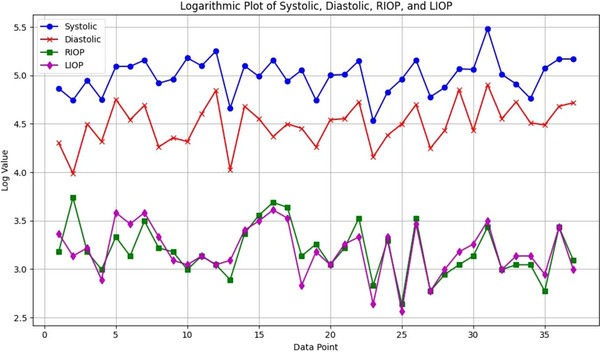
The graph shows the logarithmically scaled values of RIOP, LIOP, systolic and diastolic BP for individual dogs. Each point on the *X*‐axis represents an individual measurement, whereas the *Y*‐axis displays the values on a logarithmic scale. RIOP, right intraocular pressure; LIOP, left intraocular pressure.

A weak positive correlation was found between RIOP and SBP (*r* = 0.282, *p* = 0.045). Almost no relationship was found between RIOP and DBP (*r* = 0.129, *p* = 0.224). A significant positive correlation was found between LIOP and SBP (*r* = 0.437, *p* = 0.003). This suggests that increased SBP may also increase IOP. A significant positive correlation was also observed between DBP and LIOP (*r* = 0.347, *p* = 0.018). As expected, a high positive correlation was found between SBP and DBP (*r* = 0.764, *p* < 0.001). A significant negative correlation was found between serum P4 level and SBP (*r* = −0.445, *p* = 0.010). Similarly, a significant negative correlation was observed between P4 and DBP (*r* = −0.489, *p* = 0.005). Figure 3 displays the logarithmic scaling of RIOP, LIOP, SBP and DBP.

**FIGURE 4:**
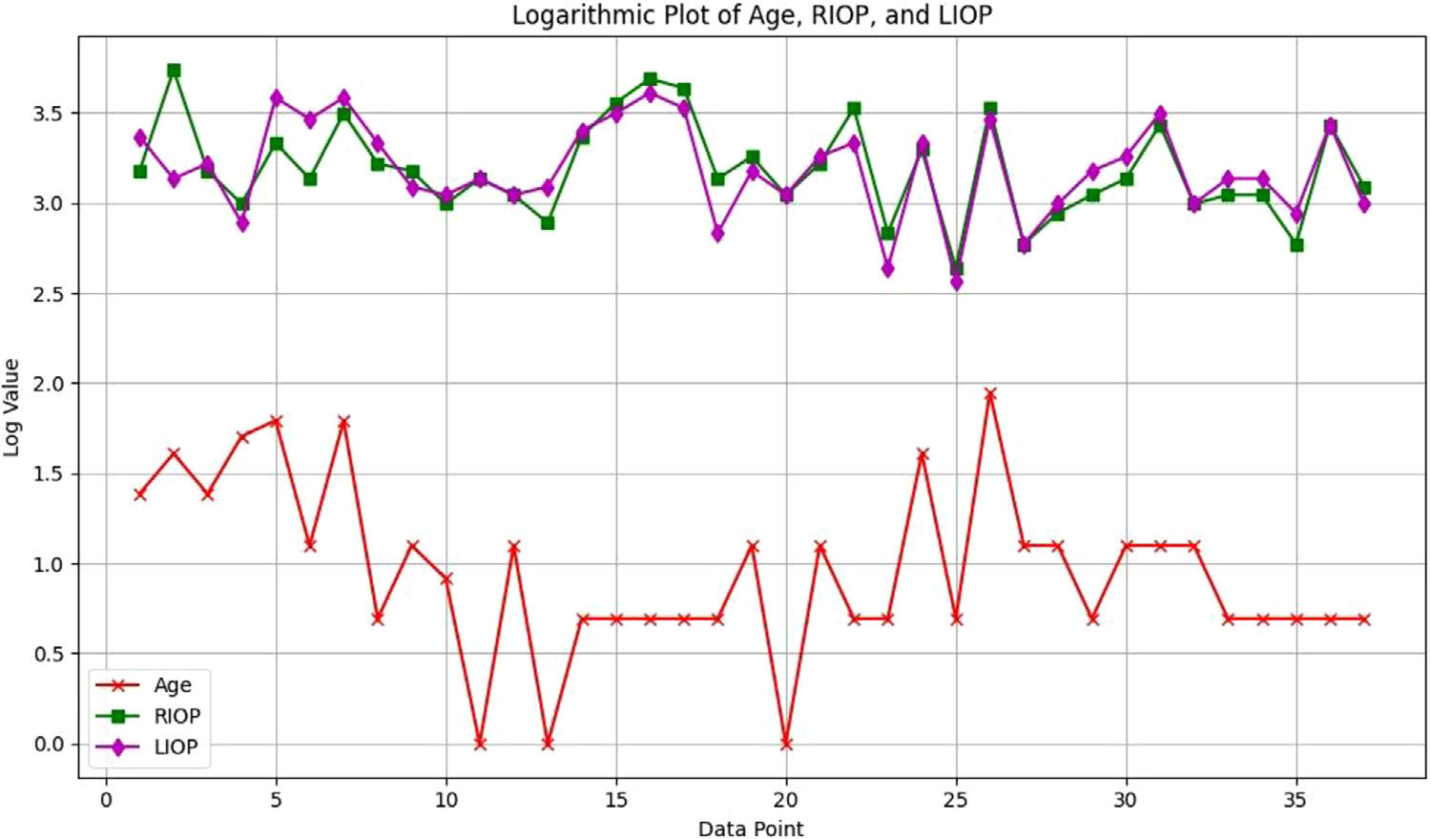
The graph shows the logarithmically scaled values of age, RIOP and LIOP for individual dogs. Each point on the *X*‐axis represents an individual measurement, whereas the *Y*‐axis displays the values on a logarithmic scale. RIOP, right intraocular pressure; LIOP, left intraocular pressure.

No significant relationship was observed between age and E2 level (*r* = −0.050, *p* = 0.369). There is a moderate positive correlation between P4 level (*r* = 0.240, *p* = 0.096). However, this result is not considered statistically significant. There is a positive correlation between age and RIOP (*r* = 0.296, *p* = 0.044), indicating that RIOP increases with increasing age. A stronger positive association was found between LIOP and age (*r* = 0.313, *p* = 0.036). There was also a very weak association between age and SBP (*r* = 0.013, *p* = 0.470) and DBP (*r* = 0.035, *p* = 0.419), which was not statistically significant (*p* > 0.05). The graph (Figure 4) displays the logarithmic transformations of age, RIOP and LIOP values.

There was a weak positive correlation between pulse rate and RIOP (*r* = 0.112, *p* = 0.207), but this relationship was not statistically significant. There was also a weak positive correlation between pulse rate and LIOP (*r* = 0.034, *p* = 0.379), which was not significant. There was a slightly greater positive correlation between pulse rate and SBP (*r* = 0.355, *p* = 0.016), indicating that SBP may increase with an increase in pulse rate. There was also no significant relationship between pulse rate and DBP (*r* = 0.022, *p* = 0.452).

A negative correlation exists between body temperature and P4 (*r* = −0.423, *p* = 0.005). This finding indicates a statistically significant relationship between the decrease in P4 levels and the increase in body temperature. The correlation between body temperature and E2 is weak (*r* = 0.005, *p* = 0.489). Weak positive correlations were identified between body temperature and RIOP (*r* = 0.085, *p* = 0.308) as well as LIOP (*r* = 0.054, *p* = 0.376), both of which were not statistically significant.

A strong positive and significant correlation exists between body weight and RIOP (*r* = 0.591, *p* < 0.001), as well as a moderate positive and significant correlation between body weight and LIOP (*r* = 0.408, *p* = 0.006). A moderate, positive and significant correlation was detected between body weight and age (*r* = 0.374, *p* = 0.006). A moderate correlation was seen between body weight and SBP (*r* = 0.362, *p* = 0.014) and a weak correlation with DBP (*r* = 0.278, *p* = 0.048).

## 4 Discussion

The relationship between serum E2 and P4 levels and IOP has become an important topic in research, particularly in the context of sex differences and hormonal influences on ocular health. E2 and P4, the primary sex hormones in dogs, appear to have a complex interaction with various physiological parameters, including IOP.

Glaucoma is marked by increased IOP, which may be intensified by hormonal changes (Pizzirani 2015; Stavinohova et al. 2015) and can be concealed by physiological aspects like pregnancy (Ozcan et al. 2024). The pathogenesis of canine glaucoma frequently entails alterations in aqueous humour dynamics that may be affected by hormone levels (Sebbag 2023; Pizzirani 2015). Significant research indicates that many pharmacological treatments and therapies designed to regulate IOP frequently engage with hormonal pathways, hence confounding the interplay between these hormones and ocular pressure (Dower et al. 2017; Desai et al. 2022).

Karaca Adıyeke et al. (2017) conducted a study that demonstrated a weak positive correlation between serum E2 levels and IOP in women with polycystic ovarian syndrome. On the basis of this, it appears that fluctuations in E2 may have an impact on IOP measurements. The study also revealed a moderate positive correlation between E2 levels and central corneal thickness, a critical factor in the regulation of IOP. This is due to the fact that corneal thickness influences the measurement of IOP and the risk of glaucoma. P4 may exert an ocular hypotensive effect, resulting in a reduction of IOP (Naderan 2018; Özcan et al. 2017; Toker et al. 2003; Newman‐Casey et al. 2014). This effect is thought to be mediated by P4 impact on aqueous humour dynamics, possibly through changes in outflow facility (Gharagozloo and Brubaker 1991; Green and Phillips 1984). The findings of this study on female dogs throughout different oestrous cycles suggest that serum E2 and P4 levels do not have a direct effect on IOP measurements. These findings suggest that, unlike some human studies reporting a direct association between fluctuations in E2 and P4 and changes in IOP, in female dogs, these reproductive hormones may modulate IOP through distinct and possibly independent physiological pathways. This may be due to species‐specific differences in oestrogen and progesterone receptor expression in ocular tissues or differing prostaglandin‐mediated regulatory mechanisms in the ciliary body. Variations in aqueous humour production or drainage sensitivity to hormonal changes may also play a role.

Several studies have found a positive association between BP and IOP (Baek et al. 2015; Reddy and Reddy 2020; Yasukawa et al. 2022; Kim et al. 2014; Janulevičienė et al. 2011; Nair and Vasudevan 2016; Foster et al. 2011). The findings of the study are in line with the literature. A positive correlation was observed between IOP and SBP measurements taken from female dogs. In human studies, proposed mechanisms for the relationship between BP and IOP include increased BP in the ciliary artery. It is emphasized that this may lead to an increase in IOP by increasing aqueous humour production (Hartmann et al. 2023; Yasukawa et al. 2022). Additionally, elevated episcleral venous pressure can impede aqueous humour outflow, resulting in higher IOP levels. Furthermore, heightened sympathetic nervous system activity has been suggested to induce changes in IOP, further contributing to this relationship (Yasukawa et al. 2022). It has been reported that the relationship between IOP and SBP may also be affected by other factors such as age, body mass index and the presence of ocular or systemic diseases (Hartmann et al. 2023; Kim et al. 2014; Yoshida et al. 2013). Our study findings support previous literature findings. The observed positive correlation between SBP and IOP in female dogs may be explained by similar mechanisms suggested in humans, such as increased aqueous humour production or vascular and autonomic factors. Additionally, it is worth noting that fluctuations in P4 and E2 levels may have the potential to influence both systemic BP and IOP. These hormonal changes could modulate vascular tone, fluid balance and autonomic nervous system activity, thereby affecting ocular physiology and contributing to the observed relationship between SBP and IOP.

P4 demonstrates a hypotensive effect, lowering SBP and DBP via multiple mechanisms (Sofuoglu et al. 2011; Reed et al. 2010; Milivojevic et al. 2016; Sammour et al. 2005). This substance antagonizes aldosterone, facilitating the excretion of sodium and chloride and promoting diuresis. Additionally, it stimulates nitric oxide production, which induces vasodilation and subsequently lowers BP. P4 also functions as a mineralocorticoid receptor antagonist, which enhances its ability to lower BP (Shi et al. 2023). This effect has been documented in both normotensive and hypertensive populations, showing reductions in SBP between 2 and 6 mmHg (Shi et al. 2023; Pang et al. 2015). The relationship is complex, with conflicting findings reported, such as an increase in SBP linked to elevated P4 levels in specific animal models (Arunogiri et al. 2021; Childs et al. 2010). This study's findings corroborate previous research indicating that P4 reduces both SBP and DBP via various mechanisms, including its functions as an aldosterone antagonist, a promoter of nitric oxide synthesis and a mineralocorticoid receptor antagonist. These findings further validate the hormone's role as a regulator of vascular function, highlighting its significance in sustaining cardiovascular stability and offering insights into its therapeutic potential for regulating BP in both normotensive and hypertensive states.

E2 and P4 concentrations fluctuate throughout the canine oestrous cycle, peaking in the follicular and luteal phases, respectively (Hong et al. 2010; Szczubiał et al. 2015). These hormones are influenced by various factors such as age, breed, body condition and reproductive conditions (Niżański et al. 2020; Frank et al. 2010; Reynaud et al. 2010; Zoltán‐Miklós et al. 2024). Obesity, weight changes and medications can further alter E2 and P4 concentrations, whereas hormonal imbalances caused by conditions such as Sertoli cell tumours or persistent Müllerian duct syndrome can lead to elevated E2 levels (Martin et al. 2006; Song et al. 2021; Park et al. 2017). In this study, no significant correlation was found between age and E2 or P4 levels in female dogs of different ages. This lack of correlation could be attributed to several factors. First, hormonal fluctuations during the oestrous cycle may confound age‐related trends, especially if dogs were at different stages of their cycle at the time of sampling. Without precise synchronization of oestrous cycle stages, changes in E2 and P4 levels may not reflect age‐related differences. Second, individual variability among dogs in hormone metabolism and regulation, influenced by genetic, breed and environmental factors, may obscure consistent age‐related patterns (Niżański et al. 2020; Frank et al. 2010). Furthermore, factors such as stress, body condition score (e.g., obesity or weight loss) or previous reproductive history may have produced variability in hormone levels (Martin et al. 2006; Daminet et al. 2003). It is also important to consider that small sample size or under‐representation of older and younger age groups may limit the detection of a statistically significant association. Furthermore, interactions between E2, P4 and other hormones, such as testosterone, may further complicate the age–hormone relationship (Song et al. 2021).

Several studies have found that IOP increases with age (Jeelani et al. 2014; Channabasappa et al. 2014), and this age‐related increase in IOP is considered a major risk factor for the development of glaucoma. The proposed mechanisms for this increase include structural changes in the eye, alterations in the ocular vasculature and potential interactions between age‐related factors and other systemic conditions (Jeelani et al. 2014; Channabasappa et al. 2014). In this study, a positive correlation was observed between age and RIOP, indicating that RIOP increases with age. A stronger positive association was found between LIOP and age, which aligns with the findings in the literature suggesting that age is a contributing factor to increased IOP. Similarly, studies have reported an increase in BP with age in healthy dogs (Sanan and Arslan 2007), with mechanisms such as increased vascular resistance and decreased arterial compliance being implicated (Laurant et al. 2004). However, in this study, there was a weak and not statistically significant association between age and SBP and DBP, which diverges from the literature. This discrepancy might be due to several factors, including the sample size, the specific population of dogs studied or the methodology used for measuring BP.

Conditions like pituitary‐dependent hyperadrenocorticism in dogs can elevate both BP and IOP, highlighting the complex interplay between these factors (Goy‐Thollot et al. 2002). However, the relationship between age, IOP and BP is not always straightforward, and other factors, such as sex, breed and underlying health conditions, may also influence these parameters (Bright and Dentino 2002). In this study, no significant relationship was observed between age and E2 levels, which may be due to hormonal fluctuations not being as prominent or influential in this specific group of dogs. There was a moderate positive relationship with P4 levels, though this was not statistically significant, further suggesting that hormonal influences might not be as influential in this study as they are in other contexts.

The examination of the impacts of reproductive hormones (oestrogens, P4, luteinizing hormone and follicle‐stimulating hormone) on the body is complicated due to the complex and multifarious actions of these hormones, both alone and combined. Oestrogen facilitates heat dissipation via peripheral vascular mechanisms that induce vasodilation, with central neuronal thermoregulatory effects that enhance cutaneous vasodilatory and sweating responses (Charkoudian and Stachenfeld 2014). Consequently, oestrogen decreases body temperature and energy expenditure, whereas P4 has the contrary influence (Cagnacci et al. 1997; Grant et al. 2020). Nonetheless, it is recognized that body temperature is influenced by factors outside hormones. Besides hormones, oxidative stress is a phenomenon intricately linked to energy expenditure and is documented to escalate during physical activity or elevated temperatures (Frisard and Ravussin 2006; McGinnis et al. 2014). This study revealed a negative association between body temperature and P4 levels. Veronesi et al. (2002) demonstrated an inverse connection between pre‐ and postnatal body temperature and plasma P4 concentrations in dogs. These findings suggest that the influence of P4 levels on the thermoregulatory region in the hypothalamus may differ between humans and dogs. Nonetheless, it is underscored that this adverse connection in canines interacts with other physiological pathways involved in the birthing process. The elevation of prostaglandin and cortisol levels, along with a reduction in P4, is significant variables influencing body temperature (Veronesi et al. 2002). Future studies investigating the association between P4 and body temperature may benefit from analysing prostaglandin and cortisol levels. This relationship may be modulated by prostaglandin F2α, cortisol and thyroid hormones, which interact with hypothalamic thermoregulatory pathways. Cortisol and prostaglandins are known to rise during parturition and have been implicated in temperature regulation, possibly enhancing or modulating the effects of P4 decline.

This study has several limitations. The lack of oestrous cycle synchronization and absence of cycle staging may have introduced variability in serum hormone levels and potentially influenced the study results. The relatively small sample size limits the statistical power to detect subtler associations. Breed‐related physiological variations were not accounted for. Future studies should include larger sample sizes, hormonal phase‐matching and hormonal receptor tissue analyses.

## 5 Conclusion

This study emphasizes the complex interaction between reproductive hormones and physiological factors in female dogs. Although E2 and P4 did not directly affect IOP, the noted correlations highlight the complex hormonal regulation of ocular and systemic functions. The strong correlation between age and IOP underscores the necessity for age‐specific monitoring in veterinary ophthalmology. The negative correlation between P4, SBP and body temperature emphasizes its essential function in vascular and thermoregulatory equilibrium. These findings offer significant insights into the hormonal dynamics influencing canine health, facilitating further research to enhance our comprehension and clinical outcomes.

## Author Contributions

**Candemir Özcan**: conceptualization, methodology, data curation, formal analysis, investigation, writing – original draft, visualization. **Tarik Safak**: conceptualization, methodology, validation, writing – review and editing, supervision. **Ali Risvanli**: validation, writing – review and editing, supervision.

## Funding

Part of this study was supported by the Kastamonu University Scientific Research Projects Coordination Unit (Project No: KÜBAP‐01/2023‐38).

## Ethics Statement

This study conducted following approval by the Kastamonu University Local Ethics Committee of Animal Experimentation (date: 01.09.2023, approval number: 2023/39).

## Conflicts of Interest

The authors declare no conflicts of interest.

## Data Availability

The data that support the findings of this study are available from the corresponding author upon reasonable request.
